# Metallic Shipwrecks and Bacteria: A Love-Hate Relationship

**DOI:** 10.3390/microorganisms13051030

**Published:** 2025-04-29

**Authors:** Laurent Urios

**Affiliations:** Université de Pau et des Pays de l’Adour, E2S UPPA, CNRS, IPREM, 64000 Pau, France; laurent.urios@univ-pau.fr; Tel.: +33-5-59-40-74-69

**Keywords:** shipwreck, bacteria, MIC, biofilm

## Abstract

For two centuries, metallic shipwrecks have been relics of the history of navigation, trade, and wars. They are also hotspots of marine biodiversity. The degradation of these shipwrecks not only threatens their environment through the release of polluting compounds, but also the reef ecosystems that have developed. Microorganisms are at the root of both degradation and reef-building, and their roles are still more hypothetical than validated. The aim of this review is to focus on the known or suggested relationships between bacteria and metallic shipwrecks and to identify issues that highlight the need for multidisciplinary studies to better understand the mechanisms at play in these ecosystems with the aim of protecting both the environment and these sites of underwater cultural and natural heritage.

## 1. Introduction

UNESCO estimates that there are over 3 million shipwrecks in the world’s oceans and seas, of which around 75% date back to the Second World War [1,2]. The cause of a shipwreck can be natural (bad weather), man-made (collision with another ship, running aground, and scuttling), mechanical, or an act of war. Over time, shipwrecks become underwater cultural heritage artifacts [3].

Among all the materials used for their construction, metallic wrecks are mainly made of ferrous alloys or aluminum for aircraft. To date, studies on aircraft wrecks have focused on corrosion and means of protection against this degradation without addressing the characterization of colonizing microorganism communities [4,5]. Consequently, this review focuses on wrecks whose structure is made of ferrous alloy.

Shipwrecks can contain hazardous substances such as explosives and hydrocarbons which, if released, can affect the environment. It is currently estimated that shipwrecks from the two world wars contain between 2.5 and 20.4 million tonnes of hydrocarbons [6]. Similarly, there are estimated to be around 1.6 million tonnes of ammunition and explosives of all types [7]. More than 8500 potentially polluting shipwrecks (PPWs) lie in the marine environment, the majority dating from the Second World War [8]. Taking into account an average corrosion rate of 0.1 mm per year, these shipwrecks, which are more than 80 years old, are extremely fragile, and the risks of destructuring, and therefore dumping, are very high [9,10]. This large number of PPWs calls for the development of classification methods, prioritization, and associated risk management strategies [11]. In addition, shipwrecks can affect living organisms in their environment when they release metals and metalloids from corrosion processes [12]. As a result, shipwrecks are considered a major source of ocean pollution by the European Parliament [2].

However, shipwrecks are not just sources of pollution. When a ship sinks, it enters an existing ecosystem, which is then disturbed. The new materials and structures that rise from the seabed will be colonized by numerous organisms [3]. They will be the physical support for a reef ecosystem that could become a real biodiversity hotspot [13].

Shipwrecks are generally involuntarily created artificial reefs that contribute to the economy through leisure activities or fishing. One of the main reasons for the attractiveness of shipwrecks is the biodiversity that concentrates on these sites [14].

Pioneer organisms colonize shipwrecks quickly after sinking, and an ecosystem is established, evolves, and stabilizes [15,16] until the shipwreck degrades and eventually disappears. During the evolution of the reef ecosystem, the stability of environmental conditions can be as influential as the nature of the materials on the succession of colonizing organisms [17].

The biodiversity observed on metallic shipwrecks can be very different from that on nearby hard substrates [18]. Similarly, community structure can differ greatly between metallic shipwrecks and artificial reefs in the same environment [19]. These structures can serve as refuges and breeding grounds for many species that find sufficient nutrients there [20,21]. But this attractiveness can also have disadvantages, such as facilitating the establishment of invasive species or the spread of organisms requiring hard substrates [22,23].

Shipwrecks that are very close to each other may have differences in their epifaunal communities. The causes are unclear, as neither the age of the wreck nor obvious abiotic factors explain the variations observed, apart from the configuration of the remains, notably their elevation in relation to the substrate, their orientation in relation to prevailing currents, and the frequency of human activities [24,25].

On soft bottoms, shipwrecks form an irregular network of hard substrates, the interconnections between which remain unclear [26]. Exchanges and relationships between wreck communities in the same geographical area must exist, but remain poorly described [27].

Given the small number of metallic shipwrecks studied in relation to the immense diversity of remains and situations, to date, each wreck could be considered a specific case and it is, therefore, necessary to increase data acquisition on these ecosystems to better understand them and guide management strategies both for their preservation and therefore, to limit pollution risks [13].

Colonization begins with the installation of microorganisms whose development on the surface of the shipwreck takes place in interaction with the materials. The aim of this review is to focus on what is known about the relationship between colonizing bacteria and metallic shipwrecks, the consequences of their development, and to identify the questions that need to be answered in order to better understand shipwreck-bacteria systems.

## 2. Shipwrecks Change Their Environment and Affect Communities

Shipwrecks physically modify their environment. Their relief and elevation affect local currents, which in turn affect water column oxygenation, nutrient transport, topography, and sediment grain size [28,29,30,31]. They also chemically modify their environment by releasing pollutants from cargo or the ship itself. The zone of influence is much wider than the perimeter of the remains. The shipwreck ecosystem extends to at least 10 times the size of the wreck itself [14].

Shipwrecks can release PCBs and trace elements (including Cu, As, Cd, Cr, Hg, Mn, Ni, Pb, and Zn) which end up in sediments, but also in organisms living on or near shipwrecks [13,32]. The pollutant plume can stretch several kilometers from the site [33].

The release of polycyclic aromatic hydrocarbons may take place slowly through seepage from reservoirs and may not be observable or significantly modify microbial communities in long-polluted environments [34], or on the contrary, affect the composition and functionalities of microbial communities [35].

Hydrocarbons, micropollutants, and heavy metals can be released simultaneously and over long periods. The study of the V-1302 *John Mahn* (WWII shipwreck) showed that 80 years after its sinking, the wreck was still influencing the chemistry and microbial ecology of its environment due to the emission of polycyclic aromatic hydrocarbons from the fuel and pollutants from the ammunitions and the ship itself [36].

Even if the sediment type was of great importance, Bolton et al. [37] showed that the frame type and country of origin of the wrecks played the most significant roles in surrounding sediments, but could not conclude on the potential impact of chemicals released from the wrecks.

In environments where iron concentration is very limited, a metallic shipwreck provides a very significant source of iron that will diffuse over a long period, encouraging the development of algae and cyanobacteria. The extent of this development disrupts that of native organisms, even going so far as to cause their death. This phenomenon, observed on coral reefs in the Pacific Ocean, has been named “black reef” after the appearance of the reefs around the wreck site [12,38,39].

## 3. Microbial Colonization

Microorganisms are the first to colonize artificial structures introduced into marine environments, and this colonization is the first step in the transformation of these structures into artificial reefs [40]. Interactions between the colonizing microorganisms and the surface of metallic shipwrecks will influence not only the integrity of the wreck, but also its evolution into an artificial reef by allowing the recruitment of other organisms [41,42].

Colonizing microorganisms form biofilms. Costerton et al. [43] proposed a definition of biofilm: “Biofilms are defined as matrix-enclosed bacterial populations adherent to each other an/or to surfaces or interfaces. This definition includes microbial aggregates and floccules”.

Biofilm formation begins within the first few hours, and maturation can take place within a few days [44,45]. The composition of the microbial community evolves over time [46]. This development strategy provides protection (toxic compounds, predators, and mechanical stress), can facilitate nutrition, communication and cooperation between cells, and recruitment of other organisms [40,47]. The matrix produced by and enveloping microorganisms is made up of exopolysaccharides, proteins, and nucleic acids, to which other organic and inorganic compounds may be added [47,48,49,50].

The development of a biofilm on the surface of the shipwreck reduces or even eliminates direct access to the metal. However, within the maturing biofilm itself, interactions with the substrate and between members of the microbial community can condition both the structuring of microorganisms according to their metabolic types, the release of compounds from the ship, and the formation of concentration gradients between the surrounding environment and the wreck surface [35,51,52,53].

## 4. Microbial Communities and Taxonomic Groups on Metallic Shipwrecks

Many studies have been carried out on coupons placed in situ to study the corrosion of steel in a marine environment. The colonization of steel coupons in marine sediment begins with an increase in the abundance of iron-oxidizing bacteria (IOB). As the biofilm develops, other organisms become established [45]. During biofilm development on the metal surface, microorganisms in the upper layers consume oxygen, thus impoverishing the deeper layers within which microaerophilic or anaerobic microorganisms can grow, such as sulfate-reducing bacteria (SRB) and methanogens [54]. The iron sulfides formed by reaction with the H_2_S produced by SRB are then oxidized by sulfur-oxidizing bacteria (SOB). During all the transformation stages, corrosive compounds are produced, which can accentuate corrosion [55]. There are very few publications describing the taxonomy of bacteria colonizing metal shipwrecks. Hamdan et al. [56] suggest that shipwrecks may function as biodiversity islands for the microbial communities that thrive there. As with macroorganisms, shipwrecks could form an irregular network of islands whose zone of influence extends several hundred meters beyond the wreck itself, with the richness and diversity of the microbiome increasing with proximity to the wreck [57]. The question of the representativeness of the sampling in relation to the total dimensions of the shipwrecks is raised. In two studies comparing the microbial communities of wreck samples with those of nearby sediments, greater similarity was shown between the communities of the different samples from the wrecks and those of their surrounding sediments and water column [42,58].

The major taxonomic groups identified from metallic shipwreck samples belong mainly to *Proteobacteria*. Mugge et al. [35] found a majority of *Alpha*-, *Epsilon*- and *Zetaproteobacteria*, among which sequences of *Mariprofundus* (*Zetaproteobacteria*) and *Sulfurimonas* (*Epsilonproteobacteria*) were very abundant, followed by *Roseobacter* (*Alphaproteobacteria*) and *Colwellia* (*Gammaproteobacteria*). In the *Pappy Lane* study [42], *Alphaproteobacteria* and *Gammaproteobacteria* were the most abundant in wreck samples. *Zetaproteobacteria* were detected in all wreck samples and in higher proportions when they were visibly corroded. They noted that *Zetaproteobacteria* can be IOB and *Planctomycetes* ammonia-oxidizing bacteria (AOB), and suggested that the latter could produce nitrogen that could be used by *Zetaproteobacteria*. They also noted that the proportion of AOB was higher in samples that were visibly corroded. Finally, Garrison and Field [59] noted that *Proteobacteria* (*Alpha*- *Beta*-, *Delta*- *Gammaproteobacteria*, and unclassified *Proteobacteria*) were in the majority (around 50% of OTUs), followed by *Bacteroidetes* and *Planctomycetes* (around 25%). They noted a similarity between wreck communities and those of immerged metallic structures. Indeed, McBeth and Emerson [45] observed the presence of *Alpha*-, *Gamma*-, *Epsilon*-, and *Zetaproteobacteria* from the first days of in situ coupon colonization, and *Deltaproteobacteria* (SRB) after a few weeks of incubation. They noted that *Zetaproteobacteria* rapidly colonized metallic surfaces in a coastal marine environment, where they were not abundant.

Other studies on steel coupons placed in a marine environment provide similar data. When monitoring community evolution, Moura et al. [60] first observed *Alteromonadaceae, Alcanivoraxaceae*, *Oceanospirillaceae*, *Vibrionaceae* (*Gammaproteobacteria*), and *Rhodobacteraceaes* (*Alphaproteobacteria*), followed by *Flavobacteriaceae* (*Flavobacteriia*). The latter could benefit from the metabolism of primo-colonizing IOB and SOB chemolithotrophs [61]. More generally, *Alpha*-, *Delta*-, and *Gammaproteobacteria* were often found, notably *Rhodobacteraceae* and *Rhizobiales*, but also *Epsilonproteobacteria* (*Sulfurimonas* and *Arcobacter*) and *Zetaproteobacteria* [46,62]. Chemolithotrophic metabolisms appear to be important among microorganisms colonizing steels. Besides metabolic pathways related to the sulfur and iron cycles, other important metabolic pathways inferred are those linked to hydrogen oxidation, arsenate reduction, and the nitrogen cycle [62]. Metabolic pathways linked to the nitrogen cycle could also affect corrosion processes [62].

The study of the microbial diversity of the *Accomac* is a special case, as it is a freshwater metallic shipwreck [58]. As already noted, the authors observed a greater similarity between the communities of the shipwreck samples than with those of the surrounding water or sediments. There is also the presence of SRB and IOB in very low proportions, which implies that even if the taxa are different from those identified in other studies, the functions are present. On the other hand, while *Pseudomonadata* is highly represented (including approximately 45% *Gammaproteobacteria* and 25% *Alphaproteobacteria*), followed by *Bacteroidota*, there is a high proportion of *Cyanobacteria*, probably due to the very shallow depth and therefore the high luminosity, which contrasts with studies on deep wrecks.

Studies carried out directly on samples from shipwrecks are very few in number compared with the multitude of studies carried out on steel coupons to address the issue of corrosion in the marine environment. The data acquired on the composition of microbial communities on shipwrecks seem to indicate a certain analogy with those on steel coupons in the marine environment. This resemblance is interesting, as the subject of corrosion is a critical one when considering the evolution of shipwrecks.

## 5. Microbial Influenced Corrosion (MIC)

Carbon steel has been used in shipbuilding since the 19th century. This inexpensive, easy-to-use material is, however, prone to corrosion. This is particularly true of a large number of metallic shipwrecks from the First and Second World Wars, which have now been lying for over a century and over eighty years, respectively [35]. Microorganism-influenced corrosion (MIC) is a phenomenon in which microorganisms accelerate corrosion directly or indirectly by creating corrosion-promoting conditions. Bacteria can create conditions that will affect corrosion, such as local pH variation, production of corrosive compounds, and reduction or oxidation of corrosion products [61].

Three categories of microorganisms are usually associated with MIC: iron-oxidizing bacteria (IOB), Iron-Reducing Bacteria (IRB), and sulfate-reducing bacteria (SRB). IOB were among the first groups of microorganisms identified as causing MIC [63,64]. IOB oxidize ferrous (Fe^2+^) to ferric (Fe^3+^). Bacterial strains belong notably to the families *Crenothricaceae*, *Sphaerotilaceae*, and *Gallionellaceae* [53] but also *Zetaproteobacteria* in aquatic environments including shipwrecks as seen previously [65]. These bacteria produce deposits made of ferric oxides and/or hydroxides [66]. Microbial iron oxide reduction contributes to MIC by the solubilization of iron compounds and the removal of iron oxides [67]. The relationship of IRB to corrosion is not simple. They can enhance corrosion or have a passivating effect [68,69]. IRB can be very diverse and can be strict or facultative anaerobes. Solid manganese and iron oxides can be used as electron acceptors for anaerobic respiration by *Shewanella* and *Geobacter* strains [70,71]. In marine environments, some SRBs can also reduce Fe^3+^ directly or indirectly by producing H_2_S, which can reduce iron oxyhydroxides to form iron sulfides [72]. Co-cultures of IOB and IRB can cause a significant loss of metal from carbon steel and greater roughening of the surface in comparison with incubations of monocultures of IOB or IRB [66].

The role of IOB in biocorrosion processes is much less well described than that of SRB. IOB could be among the first colonizers to modify environmental conditions in contact with steel, enabling other colonizers to take hold [73]. Van Landuyt et al. [36] noted a high abundance of microorganisms involved in the sulfur cycle: SRB (*Desulfovibrionia*, *Desulfobacteria*, *Desulfarculia*, and *Desulfobulbia*) and, even if it is at a very low abundance, IOB (*Mariprofundus* and *Zetaproteobacteria*).

As oxygen is consumed within the biofilm, SRB can become dominant in MIC processes. Indeed, sulfate reduction produces hydrogen and iron sulfides, which could be corrosive [74]. However, SRB activity is also dependent on the availability of nutrients and therefore, on the functioning of other types of microorganisms nearby in the biofilm: the reduction in nutrients accessible to SRB will consequently reduce their functioning and therefore, limit their effect in the corrosion process [75].

Garrison and Field [59] have attempted to define a “core steel microbiome”, as characterizing such a microbiome would help determine under which conditions steels are most susceptible or, conversely, most protected against corrosion. A core is defined as the suite of members shared among microbial consortia from similar habitats. Discovering such a core microbiome could allow us to understand the stable, consistent components within microbial assemblages [76]. Colonizing communities are affected by environmental parameters [42,45]. If they vary, the functions they perform may also be altered (carbon, nitrogen, and sulfur cycles). Members of a core microbiome can provide taxonomic and functional redundancy, and assessing the presence and proportion of each can give clues as to possible capacities to adapt to variations in environmental parameters [76]. To achieve this, community characterization must be as comprehensive as possible, as less-represented taxa may nevertheless have important roles in the functioning of these communities [77]. Somewhat surprisingly, while the metabolic types usually associated with corrosion are those related to the iron and sulfur cycles, the core steel microbiome defined by Garrison and Field [59] is predominantly made up of heterotrophic generalist taxa. Similarly, a high proportion of generalist taxa in biofilms on steels has been found to be associated with increased corrosion [35,60]. In their study of four shipwrecks including the *Titanic*, *Bismarck*, and *Britannic*, Cullimore and Johnston [78] showed that although IRB and SRB were present and sometimes quite active, heterotrophic aerobic bacteria had the highest activity rates. Biofilms on steel accumulate organic compounds that reduce or prevent other microorganisms from accessing and adhering to the metal surface and accessing this potential iron source. The degradation of these organic compounds by heterotrophic generalist microorganisms would, therefore, be an action facilitating MIC [59]. Furthermore, these generalists would be less affected by changes in their environment, giving them a competitive advantage in terms of installation and development, but also ensuring stability for the community and enabling other, more specialized microorganisms to develop [59,79].

Metallic shipwrecks corrode, and the corrosion layer sometimes takes the form of stalactites that grow over time, known as rusticles for rust-covered icicles [80]. The formation of rusticles adhering to steel is caused by communities of microorganisms that form these porous concretions containing a high proportion of metal oxides. Although the water flowing through the rusticles contains iron, the transport mechanism of corrosion products from the corrosion point to the rusticle tip is not fully elucidated, and microorganisms may be involved in particle migration [81]. The formation of rusticles and their structure and shape depend on a number of factors, including the type of steel on which they grow, and the availability of nutrients in their environment. During their development, rusticles can change in appearance and shape, while increasing in complexity, suggesting changes within the microbial community that generates rusticles [82]. This community consists mainly of SRB, acid-producing heterotrophs, denitrifiers, IRB, and IOB. The diversity of metabolisms suggests cooperative community functioning. Oxidizing conditions appear to be the predominant factor promoting rusticle growth [78]. However, at the base of these structures, under anoxic conditions and alongside SRB, methanogenic Archaea can account for more than half of the microorganisms [54].

Phototrophs (*Cyanobacteria* and microalgae) were found in high proportions in the study of the *Accomac* in freshwater [58] and of three shipwrecks in a mangrove area [83]. In the latter case, the proportions of *Cyanobacteria* were up to 35% of the sequences obtained from rusticle samples, suggesting a role in the constitution of these formations and therefore, a relationship with the metal support and its corroded forms. Microalgae have both the ability to attach to steel, causing biofouling problems, and to locally increase the concentration of dissolved oxygen through photosynthesis [84]. The question of the possible involvement of phototrophs in the corrosion processes of shipwrecks can be raised.

## 6. Protective Biofilm

Biofilm accumulation can reduce access to the metal surface and thus, protect it against abiotic generalized corrosion [51]. However, changes in environmental conditions can induce changes in the structure and composition of biofilms and thus, mitigate this protective action [85].

The production of EPS combined with the accumulation of other organic and inorganic compounds, such as calcite-forming biomineralization, constitute a so-called “hybrid” biofilm that can act as a barrier, protecting the metal surface [86]).

However, on the large surfaces that submerged structures such as metallic shipwrecks can represent, the formation of these protective biofilms may not completely and uniformly cover the surface. A random and uneven distribution of protective biofilms can then create differently oxygenated zones, producing cathodic and anodic sites and therefore, increasing corrosion at other points [87,88].

## 7. The Particular Case of Halomonas Titanicae

To our knowledge, only two studies deal with the isolation of bacteria from shipwreck samples. Price et al. [42] isolated a *Mariprofundus ferrooxydans* strain from a sample of the *Pappy Lane* shipwreck. The second study presents the isolation of a new species from the *Titanic*, and *Halomonas titanicae* BH1^T^ has become a special case. This strain was isolated from a sample of rusticle taken from the *Titanic* in 1991 [89]. Its genome was published three years later [90]. After the publication of this new species, several other isolates of the same species were isolated and used in numerous works: to date, the original publication has been cited more than a hundred times. The vast majority of publications focus on the study of steel corrosion [91,92] or copper [93], but also the behavior of *Halomonas titanicae* strains such as their adaptation to stress [94], their ability to form EPS and a biofilm [95,96]. This particular case illustrates both the key points related to the colonization of submerged metallic structures (biofilm, behavior, metabolisms, corrosion, and MIC) and the need to have a manipulable biological resource to explore in vitro which is technically very difficult to do in situ.

## 8. Environmental Changes

Natural variations in environmental conditions (tidal rhythm and freshwater inputs in estuarine areas) shape marine communities. However, periodic, chronic, or exceptional variations in water composition can exert pressures that communities must cope with. These constraints can have consequences on the degradation of shipwrecks located in affected areas, whether in anthropized coastal or deep-sea environments.

The nitrate reduction capacity is widespread in the marine environment and notably among chemolithotrophs [97,98,99]. Continental nitrate input can promote the development of nitrate-reducing bacteria (NRB). The identification in several studies of *Zetaproteobacteria* and *Epsilonproteobacteria*, sometimes in significant proportions, has led to the idea of a concentration effect of these microorganisms on metallic shipwrecks [45]. *Zetaproteobacteria* are capable of oxidizing Fe(II) and reducing nitrates, as are *Epsilonproteobacteria* [100]. The combined presence of Fe(II) and nitrates could promote the activities of these two types of bacteria, with possible consequences for corrosion.

Inputs of organic compounds such as hydrocarbons can affect the composition and functionality of biofilms on a metallic shipwreck and consequently cause accelerated corrosion [35].

## 9. Questions and Future Research Directions

The bibliography on microbial communities colonizing metallic shipwrecks is limited and complex questions still await answers. The extent of knowledge on the microbial diversity of metallic shipwrecks remains limited to a few studied sites and, to date, it appears that each wreck may be a special case. The small number of shipwrecks analyzed does not yet allow us to define a solid core microbiome specific to metallic shipwrecks. Extending the study of microbial colonization to a larger number of shipwrecks would perhaps allow us to identify a common background of microbial characteristics. In particular, exploring other freshwater sites would help to better highlight the similarities and differences with seawater sites.

A way to improve the overall understanding of wreck-biodiversity systems might be to focus more on functions rather than on taxonomy and the proportions of identified taxa. Indeed, it is the activities of colonizing microorganisms that have consequences for both the protection and degradation of materials. The effects of variations in environmental parameters that influence taxonomic diversity are perhaps counterbalanced by the functional resilience of ecosystems where the adaptive capacities and the redundancy of actors of different functions have the consequence of maintaining a functioning that would fundamentally represent a “functional core microbiome” specific to metallic shipwrecks, and why not other submerged metallic structures.

Global diversity analyses provide an image of the composition and relative proportions of taxa at generally high ranks due to the still current difficulty of having reliable identifications at the species level. They then allow metabolic inferences based on already available genomic data. But what exactly do we know about the activity of microorganisms known to be involved in the iron and sulfur cycles? Their action remains hypothetical. Is it past? Does it continue decades after the shipwreck? Measuring sulfur isotope fractionation is a technique that can detect signs of past or recent biological activity at different points in the corrosion layer, from the oldest near the metal to the most recent at the interface with the surrounding environment.

Microbial communities forming biofilms on coupons are evolving, but what about shipwrecks? Studies on the microbiome colonizing steel coupons in the marine environment have been conducted over relatively short periods of time, from a few weeks to a few months, while metallic shipwrecks lie for decades. How can we understand their evolution and that of the colonizing communities? How can we make very long-term predictions? Carrying out colonization monitoring experiments in the context of metallic shipwrecks, in parallel with the characterizations of microbiomes of shipwrecks of different ages, could help us understand how communities evolve over the long term, what the associated effects are, and perhaps be able to model the joint evolutions of communities and shipwrecks. Another experimental possibility would be to study ships that are decontaminated and sunk in order to create artificial reefs. Several studies concern the diversity of macroorganisms on such shipwrecks, but the communities of microorganisms, although first colonizers, have not been characterized from the day of immersion and then over time. These sites could make it possible to describe the first stages of colonization and then the succession of populations in situ in the medium and long term.

A few microbiological studies on shipwrecks have shown that environmental parameters affect microbial communities, including depth, salinity, hydrocarbons, and nitrates. What about other pollutants or organic compounds from human activities? Many shipwrecks are located in anthropized coastal areas. Studies taking into account these anthropogenic parameters are necessary to integrate this influence into the overall understanding of the evolution of metallic shipwrecks.

Historic wrecks have been lying for decades, and when the question of their preservation arises, they are already heavily corroded. Research based on the placement of coupons in in situ conditions aims to understand the corrosion mechanisms from their initiation. However, when they sank, the ship structures were not bare: protective paints and coatings covered them, masking most of the metal surface from the first colonizing microorganisms. To date, this aspect has not appeared in diversity studies. Using analogs of historical coatings on coupons could help address the subject. Another way would be to find traces left by colonizing microorganisms in the deepest corrosion layers, in contact with the remains of the coating, to obtain clues about the actors who initiated the process.

Not all submerged objects from historical and cultural heritage can be removed from the water and preserved in museums, especially when they are very large. Consequently, in situ protection is the only option for preserving them. Cathodic protection, which is traditionally used for ships, is a method that can be implemented on wrecks for their in situ protection [101]. Briefly, it consists of connecting sacrificial anodes to the metal object to be protected, which will modify the electrochemistry of the system created and corrode in place of the protected object. The authors of [102] summarized the numerous studies dealing with the effects of cathodic protection on MIC. This synthesis showed contrasting and sometimes contradictory effects depending on whether the tests were carried out in the laboratory or in situ, and depending on the microorganisms studied. What are the consequences of the depolarization of a metallic wreck on the microbial community colonizing it? To our knowledge, no study has been published on this issue to date, meaning that this problem linked to heritage conservation remains to be explored.

Accessing wrecks lying on the seabed can be much more difficult than for historical sites on land. They can be located at great, even abyssal, depths, requiring specific and expensive technical resources (specific vessels, professional divers, submarines, or ROVs). Historic wrecks can also be protected. As such, authorizations are required to conduct experiments on them. These authorizations depend on national organizations, and the procedures can be long and complex. Sampling fragments, even small, from wrecks is necessarily destructive. Placing coupons on wrecks is an alternative for studying in situ processes. This can allow us to track the mechanisms of material evolution without compromising the integrity of the wrecks. However, this substitute makes it impossible to analyze the past evolution of material-biodiversity systems over decades. Similarly, monitoring these coupons over the very long term (up to several decades) is difficult. All these factors increase the difficulty of carrying out studies and constitute obstacles to the development of this research theme. They demonstrate the need to involve in projects those who are able to provide the technical means and regulatory authorizations, while knowing that the discoveries and knowledge resulting from the work will be beneficial to all, thus showing them the value of developing cooperative multidisciplinary approaches.

## 10. Conclusions

Metallic shipwrecks could be considered as long-term corrosion experiments that cannot be reproduced in vitro [81]. Each wreck may be considered as a special case. Indeed, the number of metallic shipwrecks studied remains very limited compared to hundreds of thousands, even millions of others. In addition, the composition of the microbial communities of the sites studied varies between sites and even depending on the sampling points of the same wreck. These variations appear to be due to environmental parameters such as exposure to currents and tides, depth, salinity, or even the effect of hydrocarbons [35,42,58,59,62]. It is difficult to consider this corpus of study as a homogeneous group representative of all metallic shipwrecks. Consequently, it raises the problem of fundamental global understanding, extrapolation, and formulation of generalizable solutions to slow down their degradation while preserving the reef ecosystems of which they are, from the outset, a vital element for their development and survival.

Metallic shipwrecks are attractive to certain bacterial taxonomic groups whose development on their surface has consequences both in the short term (initiation of the reef ecosystem) and in the long term (protection or accentuation of corrosion). This could be the case for *Zetaproteobacteria*, for which submerged metallic structures such as wrecks could play a concentrating role. These bacteria, known as Fe(II)-oxidizers, find favorable conditions for their development on these sites. The future of metallic shipwrecks, therefore, depends in particular on colonization by these microorganisms, but the reverse is true: as the shipwrecks degrade, disintegrate, and disappear, the reef ecosystem is threatened and eventually disappears as well. Like other colonizing macroorganisms, microorganisms then gradually lose their development support both as a surface and as a source of useful compounds.

The isolation of new strains and the subsequent study of both their genomes and their actual metabolic capacities are limited to a few cases. However, these cases have allowed the acquisition of important data. A significant part of bacterial diversity in general, and colonizing metallic shipwrecks in particular, remains to be explored, judging by the numerous uncultured taxa revealed by metagenomic studies. Isolating and studying the physiology and metabolisms of new strains requires a lot of time and effort, but it can provide information that allows us to validate or not inferences based solely on sequencing data.

Marine structures are expected to increase in number and area by at least 23% globally over the next 10 years [103]. It is important to document the evolution of the seabed landscape in order to understand the effects on ecology before the arrival of new structures [62]. As submerged structures are supports and refuges for marine organisms, the increase in these structures raises questions about their effects on biodiversity and ecosystem functioning. The study of biofilms is, therefore, necessary to address these questions. The role of microorganisms colonizing submerged metallic structures is crucial in several ways, both in potential protection and in degradation mechanisms. The phylogenetic and functional diversity of biofilms that provides them with resistance, adaptation, or resilience to changes can be both beneficial and problematic [88]. Understanding the composition and dynamics of communities within these complex biofilms appears necessary to imagine preservation solutions for both metallic shipwrecks and other submerged structures. In particular, the use of cathodic protection deserves to be studied from the design stage of in situ protection projects using this method. Collaboration between physical chemists and biologists is necessary in order to monitor over time the effectiveness of the protocol used and the potential effects on colonizing diversity.

Underwater cultural heritage offers a vast network of ecological experimentation and monitoring sites that can contribute to expanding our collective understanding of the ecological functions of submerged structures [3]. Collaborations between archeologists and biologists are necessary to achieve an understanding of the processes that occur on and around shipwrecks. Knowledge of shipwreck ecology has implications for ecology but also for archeology and vice versa. Furthermore, collaboration with other disciplines interested in environmental conditions and physicochemical processes (oceanography, physics, and chemistry) is necessary to better understand the functioning of wreck-living systems [104]. The subject is so vast and complex that its exploration can only truly progress within the framework of such multidisciplinary collaborations.
